# Preventive Effect of Nutrition Support on Peroneal Neuropathy in Cancer Patients

**DOI:** 10.3389/fnut.2018.00139

**Published:** 2019-01-08

**Authors:** Hiroyuki Fukuda, Aiko Yamashita, Toru Imai, Hiromi Tsumaki, Namiko Nagata, Hiroshi Ishikawa, Masahiro Niihara, Yasuhiro Tsubosa, Yusuke Onozawa

**Affiliations:** ^1^Division of Neurology, Shizuoka Cancer Center Hospital, Shizuoka, Japan; ^2^Division of Nutrition, Shizuoka Cancer Center Hospital, Shizuoka, Japan; ^3^Clinical Research Promotion Unit, Shizuoka Cancer Center Hospital, Shizuoka, Japan; ^4^Department of Nursing, Shizuoka Cancer Center Hospital, Shizuoka, Japan; ^5^Department of Pharmacy, Shizuoka Cancer Center Hospital, Shizuoka, Japan; ^6^Division of Esophageal Surgery, Shizuoka Cancer Center Hospital, Shizuoka, Japan; ^7^Division of Clinical Oncology, Shizuoka Cancer Center Hospital, Shizuoka, Japan

**Keywords:** cancer, nutrition, nutrition support, occurrence, peroneal, peroneal neuropathy, weight loss

## Abstract

**Background:** The occurrence of peroneal neuropathy was reported to be higher in cancer patients than in non-cancer patients. Cancer patients should have their nutritional intake carefully managed in order to minimize weight loss and avoid the occurrence of peroneal neuropathy. However, the effect of nutrition support on the prevention of peroneal neuropathy is not understood.

**Aims:** The present study aimed to assess epidemiologically the effect of nutrition support on the occurrence of peroneal neuropathy in cancer patients.

**Methods:** We performed a retrospective case-control study in 178,597 cancer patients admitted to Shizuoka Cancer Center Hospital from 2002 to 2017. The rates of peroneal neuropathy were assessed between the 8-year period before nutrition support started and the 7-year period during which nutrition support performed.

**Results:** Twenty-nine and 14 cases of peroneal neuropathy occurred in the periods without and with nutrition support, respectively. Compared with the period without nutrition support, the risk of peroneal neuropathy decreased by 62% during the period with nutrition support (odds ratio, 0.38; 95% confidence interval, 0.18–0.74; *P* < 0.05).

**Conclusion:** Our study suggests the preventive effects of nutrition support on peroneal neuropathy in cancer patients.

## Introduction

Cancer patients are at a high risk of weight loss, which affects a patient's response to treatment as well as their survival and quality of life (QOL) (1–3). The importance of providing support to help maintain the nutritional status and QOL has been emphasized in cancer patients (4).

Peroneal neuropathy is the most common entrapment neuropathy of the lower extremities in clinical practice (5). Numerous etiologies have been identified, and the most common cause is compression around the fibular head. Habitual leg crossing, decubitus position and prolonged bed rest are related to the performance status of patients and contribute to compression around the fibular head. The peroneal nerve may be easily compressed when the adipose tissue around the fibular head becomes thin due to weight loss (6–8). The use of pneumatic compression devices for preventing deep venous thrombosis during surgery also increases the risk of peroneal neuropathy (9).

The occurrence of peroneal neuropathy was reported to be higher in cancer patients (0.64%) than in non-cancer patients (0.07%). The crude relative risk was 8.6, and the relative risk after correction for differences in age and sex was 3.4 between cancer and non-cancer patients (10). The frequency of serious weight loss in cancer patients has been regarded as a main factor underlying the difference in the occurrence (10–12).

If nutrition support can reduce the quantity of weight loss in cancer patients, the occurrence of peroneal neuropathy should also decrease. However, the extent to which the risk of peroneal neuropathy can be reduced by nutrition support is unclear, although the efficacy of nutritional therapy on the QOL in cancer patients has been addressed (13, 14). The present study aimed to assess the effect of nutrition support on the occurrence of peroneal neuropathy in cancer patients using a single cancer institute database.

## Methods

This study was a retrospective case-control study carried out at Shizuoka Cancer Center Hospital in Japan. The hospital database of consecutive patients admitted to the institute from September 2002 to March 2017 was reviewed. The observation period was divided into the following two intervals: the first period (8 years before nutrition support was started) and the second period (7 years when nutrition support was active). The age and gender of all admitted patients were collected. To elucidate chronological changes in the characteristics of the admitted patients, the numbers of patients who received surgery, chemotherapy and radiation therapy as well as the average length of hospitalization and the number of deceased patients were collected. The data were analyzed by Fisher's exact test with a two-sided *P*-value of < 0.05 to indicate statistical significance.

The clinical documents of the patients tentatively diagnosed with peroneal neuropathy in the hospital database were evaluated retrospectively. The inclusion criteria were muscle weakness of the tibialis anterior and extensor hallucis longus associated with sensory impairment of the toes. Agreement on the diagnosis by more than two physicians was confirmed. In the cases of sensory predominant disturbance without definite motor deficits, a nerve conduction study and electromyography were required. Patients were excluded if they had myopathy, polyneuropathy, lumbar radiculopathy, sciatic neuropathy, brain metastasis, history of surgical operation adjacent the peroneal nerve, or other diseases. The type of tumor and possible causes in peroneal neuropathy patients was also assessed.

The nutrition database at our hospital between September 2002 and March 2017 was retrospectively reviewed. The details of activities of nutrition support and the number of patients who received nutritional intervention were assessed.

We used the odds ratio to analyze the change in the peroneal neuropathy incidence during the two periods. To adjust for potential biases by confounders, we considered correcting for differences in the age or gender distribution. Fisher's exact test was used, and a two-sided *P*-value of < 0.05 was considered to indicate statistical significance. All statistical analyses were conducted using the R statistical software program, ver. 3.4.0 (R foundation, Vienna, Austria).

All procedures were in accordance with the ethical standards of the responsible committee on human experimentation (institutional and national) and complied with the 1964 Declaration of Helsinki as well as later versions. The study protocol was reviewed and approved by the Ethical Review Committee of Shizuoka Cancer Center Hospital (approval number: 29-J96-29-1-3). Informed consent was obtained in the form of opt-out on the bulletin board and web-site of the hospital and guaranteed the opportunity for research subjects to refuse participation in research.

## Results

A total of 178,597 patients (74,753 women) were involved in this study. The patient characteristics are summarized in Table 1. The average of age during the second period was higher by 1.7 than that of the first period (*P* < 0.00001). The gender ratio was similar between the periods (*P* = 0.62). The numbers of admitted patients, surgeries, chemotherapy, and radiation therapy as well as the number of deceased patients were higher and the average length of hospitalization was shorter in the second period than in the first period (Table 1).

**Table 1:** Characteristics of patients admitted in the first period (from September 2002 to March 2010) and the second period (from April 2010 to March 2017).

	**First period (*n* = 78,374)**	**Second period (*n* = 100,223)**	***P***
Age (years)	62.8 ± 13.8	64.5 ±14.0	< 0.00001
Women (%)	32,760 (42)	41,993 (42)	0.62
Surgery (*n*/year)	2927.5 ± 861.5	4279.1 ± 338.7	0.037
Chemotherapy (*n*/year)	2281.6 ± 794.7	3173.0 ± 236.9	0.009
Radiation therapy (*n*/year)	1379.5 ± 377.8	1781.7 ± 112.0	0.009
Average length of hospitalization (day)	15.9 ± 3.1	13.1 ± 0.6	0.023
Deceased patients (*n*/year)	804.5 ± 285.3	1112.1 ± 76.2	0.001

A total of 66 patients were tentatively diagnosed with peroneal neuropathy from September 2002 to March 2017. An electrodiagnostic study was not performed in almost half of the cases because of their serious conditions. Twenty-three cases were excluded for the following reasons: no definite neuropathy or a poor study (*n* = 7), operation adjacent to the peroneal nerve (*n* = 5), lumbar radiculopathy (*n* = 3), history of peroneal neuropathy (*n* = 3), myotonic dystrophy (*n* = 2), sciatic neuropathy (*n* = 1), brain tumor (*n* = 1), and necrosis of the foot (*n* = 1). Consequently, 43 cases of peroneal neuropathy were identified (12 women), and the median age was 65 (10 to 90) years. The prevalence of peroneal neuropathy was 0.04% (29 cases) in the first period, 0.01% (14 cases) in the second period, and 0.02% in the total observation period (Table 2). The most common cancers were pulmonary (*n* = 8), followed by hepatobiliary tumors (*n* = 7), pancreas tumors (*n* = 7), head and neck and esophagus tumors (*n* = 5), and others (*n* = 16). For most types of tumor, the incidence of neuropathy was lower in the second period than in the first period (Table 3). Peroneal neuropathy associated with surgery, leg crossing, and a bedridden state did not differ markedly between the two periods compared with other factors (Table 4).

**Table 2:** Numbers (percentages) of patients with and without peroneal neuropathy in the first period (from September 2002 to March 2010) and the second period (from April 2010 to March 2017).

	**Peroneal neuropathy**	**No peroneal neuropathy**	**Total**
First period	29 (0.04)	78,345	78,374
Second period	14 (0.01)	100,209	100,223
Total	43 (0.02)	178,554	178,597

**Table 3:** The types of tumor in the patients with peroneal neuropathy in the first period (from September 2002 to March 2010) and second period (from April 2010 to March 2017).

**Tumor**	**First period**	**Second period**	**Total**
Pulmonary	5	3	8
Hepatobiliary	5	2	7
Pancreas	4	3	7
Head and neck, esophagus	4	1	5
Hematological	4	0	4
Colon	2	1	3
Stomach	2	1	3
Urology	0	2	2
Brain	1	0	1
Skin	1	0	1
Bone	1	0	1
Ovary	0	1	1
Total	29	14	43

**Table 4:** Characteristics and contributing factors in patients with peroneal neuropathy in the first period (from September 2002 to March 2010) and the second period (from April 2010 to March 2017).

	**First period (*N* = 29)**	**Second period (*N* = 14)**	**Total (*N* = 43)**
Age (years)	61.4 ± 16.3	69.6 ± 12.0	64.3 ± 15.3
Women	6	2	8
Post-operative	7	6	13
Diabetes	8	3	11
Alcohol	10	3	13
Weight loss>5 kg	20	8	28
Albumin < 3.5 g/dl	14	11	25
Bilateral	4	1	5
Leg crossing	7	5	12
Bedridden	7	6	13

Nutritionists provided nutritional counseling to cancer patients during both periods. The main purpose of the counseling during the first period, however, was the management of dietary changes in order to tailor the diet to individual habits and education to avoid damping syndrome in patients after gastric surgery. The importance of nutrition support in cancer patients was poorly recognized at the beginning of the twenty first century in Japan. As such, organized efforts to try to improve the nutritional status of cancer patients was not performed at our hospital at that time.

A multi-disciplinary nutrition support team composed of nutritionists, physicians, nurses, and pharmacists was finally started at our hospital in April 2010. Patients consulted with the nutrition support team after screening for nutritional risk, as determined by an inadequate nutritional intake for over 2 weeks, >5% weight loss for a month or >10% weight loss for 6 months and hypoalbuminemia (< 3.0 g/dl). All patients with head and neck cancer and esophageal cancer were consulted, as most with these cancers had difficulty with their oral intake. The assessment of the nutritional state and nutritional counseling were the first interventions provided to each patient. The body mass index, body composition, and total energy expenditure (sum of the resting energy expenditure plus activity-associated energy expenditure) were calculated. The white cell counts, C-reactive protein, albumin, and prealbumin as biomarkers for nutrition were measured. The volume, contents, and route of nutrition as well as the measures against nausea, vomiting, and diarrhea were discussed at a conference twice a week. Oral nutrition support consisted of an increased size of meals and the use of drink supplements. Nutritional supplements through a nasogastric tube or gastrostomy were also considered when patients were unable to take their meals orally. If adequate oral or enteral nutrition was not possible, parenteral nutrition was advocated. The team also helped educate the patients, family members, and medical staff through conferences in order to underscore the importance of the nutritional intake at home and in the hospital. The average number of nutritional interventions was 842.3 (571 to 1,119) per year. This accounted for 0.6% of all admitted patients in the second period.

None of the patients who received nutritional intervention developed peroneal neuropathy, and none of the 14 patients with peroneal neuropathy received nutrition support in the second period (Table 5). The neuropathy occurred just after the operation in 6 of 14 patients. Most surgical patients in our hospital were routinely managed by a clinical pathway that regulate the contents and volumes of nutrition suitable for each type of cancer. Therefore, many surgical patients except for those with head and neck cancer and esophageal cancer did not receive nutrition support but did receive an individual-based nutrition counseling. Five patients with end-stage cancer were deemed not indicated for nutrition support. Two patients receiving chemotherapy did not receive nutrition support despite it being suitable for them. The main reason for the lack of nutrition support in these patients was that the attending physicians were not interested in their nutrition care. One patient was not assigned nutrition support because he was before encountered before the diagnosis of cancer was confirmed.

**Table 5:** Numbers of patients with and without peroneal neuropathy in the second period (from April 2010 to March 2017).

	**Peroneal neuropathy**	**No peroneal neuropathy**	**Total**
Nutrition support	0	5,896	5,896
No nutrition support	14	94,313	94,327
Total	14	100,209	100,223

The risk of peroneal neuropathy decreased by 62% during the period with nutrition support (odds ratio, 0.38; 95% confidence interval, 0.18–0.74; *P* < 0.05) compared with the period without nutrition support. Differences were corrected in the distribution of age but not in that of gender.

## Discussion

This study epidemiologically investigated the effect of nutrition support on peroneal neuropathy in cancer patients. The results showed that nutrition support was associated with a significant reduction in the occurrence of peroneal neuropathy.

Previous studies have shown the frequency and possible causes of the neuropathy in cancer patients and stressed close monitoring in order to avoid weight loss and pressure around the peroneal head (10–12, 15). However, whether or not excluding these possible causes can prevent neuropathy has not been evaluated. Our hospital was founded in 2002 and is regarded as the highest-activity cancer center in Japan. The numbers of admitted patients, surgeries, chemotherapy, radiation therapy, and deceased patients have been increasing as a consequence of the expansion of our hospital bed capacity. The categories and distribution of the admitted cancer patients have not changed markedly over the past 15 years. The shortened average length of hospitalizations, which has been the shortest in recent years among main cancer centers in Japan, seems to indicate an increase in acute-phase patients. The increased in the number of deceased patients may suggest an increase in number of cachexic patients.

To investigate the therapeutic effect on patients, a survey must be conducted in a patient group with the same disease or in the same specific state. It thus seems difficult to gather and analyze data on body weight change due to nutritional support in a group of patients placed in various situations with different types of cancer. The rate of peroneal neuropathy was as low as 0.02% in our study and 0.64% in a previous report (10). To observe changes in the occurrence of peroneal neuropathy, a large number of patient groups is necessary, and the number of subjects may be insufficient in a group of patients placed in a particular situation. For example, only 4 cases of peroneal neuropathy were reported among 450 head and neck cancer patients that were at high risk of severe weight loss and peroneal neuropathy (16). In other words, it seems impossible to study the influence of nutritional support on weight loss and the change in the occurrence of peroneal neuropathy simultaneously. Using a case-control study to observe the effects of exposure to infrequent diseases is widely accepted as an epidemiological study. We thus believe that it is appropriate to perform a case-control study to assess the influence of nutritional support on the occurrence of peroneal neuropathy for many patients in various situations with different types of cancer.

The prevalence of peroneal neuropathy in cancer patients was 0.02% in our study and 0.64% in a previous study (10). This difference seemed to be attributed to different customs in Japan and the Netherlands, where the previous study was conducted. The Japanese (particularly older people) do not often cross their legs, as sitting down with legs crossed is considered rude in Japanese culture. Habitual leg crossing is widely regarded as a major cause of compression around the fibular head (6–8).

The occurrence of peroneal neuropathy in cancer patients is reported more often in men and in older patients than in women and younger patients (10, 11). We corrected for differences in the age but not in the gender in the present study, as the average of age was different while the gender ratio was similar between the two periods.

A systematic review and meta-analysis observed that dietary counseling did not result in a statistically significant improvement in the QOL of cancer patients (13); however, ESPEN guideline suggesting that nutritional counseling may be justified in select patients suffering from an especially poor oral intake and weight loss (17). Another systematic review and meta-analysis reported the efficacy of oral nutritional interventions on some aspects of the QOL (emotional functioning, dyspnea, loss of appetite, and the global QOL) in cancer patients (14). However, a reliable effect of nutritional intervention on the QOL in cancer patients could not been found, and further investigations are thought to be necessary (17).

Considering the number of members on our team, it would be impossible to implement nutrition support for all admitted patients, and the number of cases in which it is implemented must be limited to some extent. However, several serious issues that should be resolved remain. As shown in Table 4, the numbers of cases of peroneal neuropathy associated with surgery, leg crossing, and bedridden state showed no marked decreased between the two periods compared with other factors. Most surgical patients except for those with head and neck cancer or esophageal cancer did not receive nutrition support and have been managed via an appropriate clinical pathway. Over 1,000 patients died every year, and most patients with end-stage cancer are not indicated to nutrition support. Many patients with end-stage disease should be recognized to be in a bedridden state and to have cachexia, which can easily cause the neuropathy. Some physicians do not seem to be interested in nutrition care. To increase the number of patients receiving nutrition support which could reduce the occurrence of peroneal neuropathy, we must try to administer nutrition support to more surgical and cachexic patients and to encourage physicians to pay more attention to nutrition care.

The results of our study showed a significant reduction in the occurrence of peroneal neuropathy in the admitted cancer patients, despite the small percentage of cases with nutrition intervention. This decreasing tendency was seen in most types of tumor. We considered that patients with a high risk of the neuropathy were effectively selected by the screening and received appropriate nutritional intervention. In addition, nutritional intervention in all patients with head and neck cancer and esophageal cancer seemed to be effective in preventing neuropathy, as the patients with these cancers were at risk of severe weight loss and peroneal neuropathy (16).

In conclusion, we found that the occurrence of peroneal neuropathy in cancer patients was significantly reduced during the performance of nutrition support. To our knowledge, no reports have described the relationship between the efficacy of nutrition support and the occurrence of peroneal neuropathy in cancer and non-cancer patients. This is the first report to describe the preventive effects of nutrition support on the occurrence of peroneal neuropathy. These results may encourage further research regarding the provision of nutrition support in cancer patients.

## Limitations

We acknowledge there are several limitations associated with our study. First, the results of this study are based on a retrospective analysis at a single institute, and no prospective cohorts were included. Furthermore, we focused on investigating the data through a case-control study, not on analyzing the direct effects of nutrition support on cancer patients. Second, we were unable to perform an electrodiagnostic study for the diagnosis of peroneal neuropathy in all patients. Third, we reduced the bias by estimates due to age differences between the two periods, but biases from unobserved differences may still have occurred. However, despite these limitations, this study provides an overview of the risk of peroneal neuropathy resulting from nutrition support.

## Author Contributions

HF, AY, HT, NN, HI, MN, YT and YO designed the study, collected the data, and contributed to the data analysis, results interpretation, and the manuscript preparation. TI contributed to the data analysis, results interpretation, and the manuscript preparation. All of the authors contributed to the review, editing, and approval of the final manuscript.

### Conflict of Interest Statement

The authors declare that the research was conducted in the absence of any commercial or financial relationships that could be construed as a potential conflict of interest.
